# Migrant Workers in Malaysia: Current Implications of Sociodemographic and Environmental Characteristics in the Transmission of Intestinal Parasitic Infections

**DOI:** 10.1371/journal.pntd.0005110

**Published:** 2016-11-02

**Authors:** Norhidayu Sahimin, Yvonne A. L. Lim, Farnaza Ariffin, Jerzy M. Behnke, John W. Lewis, Siti Nursheena Mohd Zain

**Affiliations:** 1 Institute of Biological Science, Faculty of Science, University of Malaya, Kuala Lumpur, Malaysia; 2 Department of Parasitology, Faculty of Medicine, University of Malaya, Kuala Lumpur, Malaysia; 3 Primary Care Medicine, Faculty of Medicine, University Teknologi Mara Sungai Buloh Campus, Selangor, Malaysia; 4 School of Life Sciences, University of Nottingham, University Park, Nottingham, United Kingdom; 5 School of Biological Sciences, University of London, Egham, Surrey, United Kingdom; Universidad Nacional Autónoma de México, MEXICO

## Abstract

A cross-sectional study of intestinal parasitic infections amongst migrant workers in Malaysia was conducted. A total of 388 workers were recruited from five sectors including manufacturing, construction, plantation, domestic and food services. The majority were recruited from Indonesia (n = 167, 43.3%), followed by Nepal (n = 81, 20.9%), Bangladesh (n = 70, 18%), India (n = 47, 12.1%) and Myanmar (n = 23, 5.9.2%). A total of four nematode species (*Ascaris lumbricoides*, *Trichuris trichiura*, *Enterobius vermicularis* and hookworms), one cestode (*Hymenolepis nana*) and three protozoan species (*Entamoeba histolytica/dispar*, *Giardia* sp. and *Cryptosporidium* spp.) were identified. High prevalence of infections with *A*. *lumbricoides* (43.3%) was recorded followed by hookworms (13.1%), *E*. *histolytica/dispar* (11.6%), *Giardia* sp. (10.8%), *T*. *trichura* (9.5%), *Cryptosporodium* spp. (3.1%), *H*. *nana* (1.8%) and *E*. *vermicularis* (0.5%). Infections were significantly influenced by socio-demographic (nationality), and environmental characteristics (length of working years in the country, employment sector and educational level). Up to 84.0% of migrant workers from Nepal and 83.0% from India were infected with intestinal parasites, with the ascarid nematode *A*. *lumbricoides* occurring in 72.8% of the Nepalese and 68.1% of the Indian population. In addition, workers with an employment history of less than a year or newly arrived in Malaysia were most likely to show high levels of infection as prevalence of workers infected with *A*. *lumbricoides* was reduced from 58.2% to 35.4% following a year’s residence. These findings suggest that improvement is warranted in public health and should include mandatory medical screening upon entry into the country.

## Author Summary

Neglected intestinal parasitic infections (IPIs) such as soil-transmitted helminthes (STH) have been recognized as one of the main causes of illnesses especially among disadvantaged communities. The last survey of parasitic infections among migrant workers in Malaysia was conducted more than a decade ago. Although it provided useful methodological enquiries, the accrued data were not designed for policy recommendations. Over the last decade, the number of migrant workers has grown exponentially. There is an acute need for accurate information on the epidemiology of parasitic infections and infectious diseases especially as they affect urban communities in Malaysia. A particular gap has been identified in understanding the presence, persistence and epidemiology of infections among longer-term residents and immigrants who have settled in Malaysia. Hence, there is a need for a comprehensive study to establish the composition and transmission of parasitic infections in these communities with a view to developing effective methods of prevention, control and treatment of these infections. Therefore, this study is timely in adopting a scientific approach to address an important public health problem and to provide conclusions that can inform the design of effective public health policies.

## Introduction

Mass migration from less developed to more developed countries have created a shift in the global population. Urbanization and extensive industrialization of developing nations have resulted in millions of migrants travelling to major urban cities around the globe to join the expanding workforces. The International Labor Organization (ILO) estimates that there are approximately 232 million international migrant workers worldwide. Globalization, demographic shifts, conflicts, income inequalities and climate change are some of the influences that drive workers and their families to cross borders in search of better employment and security [1]. In Malaysia, the robust economic growth of the different sectors has led to the mushrooming of small to large enterprises requiring high demand of a low-skilled workforce primarily in sectors such as construction, domestic and food services, manufacturing and plantation. This has attracted many to flock to the country both legally and illegally [2,3] from South East Asian (Indonesia, Cambodia, Vietnam, the Philippines and Myanmar) and West Asian countries (Nepal, India and Bangladesh) [2,3] where endemic infections are very much prevalent and most likely to pose public health problems to the local community [4,5,6,7].

Malaysia is a middle-income country whose economy has transformed into an emerging multi-sector economy and since the 1970s it has been facilitated largely by imported migrant workers. Malaysia has a higher standard of living compared with other neighboring countries in the South East Asian and West Asian region. A total of 74.7% of the population in Malaysia has undergone urbanization with 2.66% annual rate of change (2010–2015) [8]. Access to sanitation facilities in Malaysia has improved also in both urban and rural areas for up to 96.0% of the population. Meanwhile drinking water sources have improved for up to 98.2% of the population [8]. The percentage of the population in Malaysia still living below the poverty line is 3.8%, considerably lower than that of other nationalities recruited in the present study. Myanmar has reported the highest percentage of its population living below the poverty line (32.7%), followed by Bangladesh (31.5%), India (29.8%), Nepal (25.2%), Vietnam (11.3%) and Indonesia (11.3%). The push factors for migration include poor remuneration and slim employment opportunities in their home country. Meanwhile the main factors for choosing Malaysia as a destination country are perceived to be abundant opportunities, high wage levels and attractive job offers [9].

Neglected intestinal parasitic infections (IPIs) such as soil-transmitted helminthes (STH) have been recognized as one of the main causes of illnesses especially among disadvantaged communities [10, 11]. According to the World Health Organization (WHO), STH have been identified as one of 17 neglected tropical diseases, with more than 1.5 billion people or 24% of the world’s population infected [12] with roundworm (*Ascaris lumbricoides*), whipworm (*Trichuris trichiura*) and hookworms (*Necator americanus* and *Ancylostoma duodenale*) primarily through soil contaminated by human feces. These infections can cause anemia, vitamin A deficiency, stunted growth, malnutrition, intestinal obstruction and impaired development [13]. Mild infections in adults normally are asymptomatic however in serious ascariasis infections symptoms include shortness of breath, coughing/gagging/whizzing, irregular stool, abdominal pain, nausea and vomiting. While symptoms of heavy hookworm infections include itchy rash, blood in stool and abdominal pain and infections with trichuriasis include abdominal pain, inflammatory bowel and rectal prolapse. It is estimated currently up to 800 million people are infected with *A*. *lumbricoides*, 600 million people with *T*. *trichiura* and 600 million with hookworms [10,14,15].

In addition, common human intestinal protozoan infections such as *Entamoeba histolytica/ dispar*, *Giardia duodenalis* and *Cryptosporidium* spp. [10, 15] are also widespread. It is estimated that there are 50 million cases of invasive *E*. *histolytica* disease each year, resulting in as many as 100,000 deaths. In several parts of the world, *Entamoeba* infection affects 50% of the population especially in areas of Central and South America, Africa, and Asia [16]. Whilst *G*. *duodenalis*, a parasite that is frequently associated with cases of diarrheal disease throughout the world, affects approximately 200 million people worldwide [17, 18]. On the other hand, *Cryptosporidium* spp. infection has been reported in every region of the United States [19] and throughout the world, with approximately 4% of people in developed countries infected [20]. Intestinal protozoan infections are spread by the fecal-oral route, so infections are widespread particularly in areas with inadequate sanitation and water treatment [10,15,21,22].

There is continuous migration of populations from rural to urban areas as well as mass influx of immigrants from neighboring countries to big cities. This sudden influx of people has contributed to the mushrooming of numerous mega urban slums where the environment is conducive for the transmission of intestinal pathogens [11]. Studies on parasitic infections amongst migrant workers have been conducted worldwide particularly in Asia, for example in Thailand [23,24,25], Taiwan [26,27,28,29], Taipei [30] and in the middle east primarily in the Kingdom of Saudi Arabia; Abha district [31], Riyadh [32], Al-Khobar [33], Makkah [34], Al-Baha [35] and Medina [7]. In Qatar, Abu-Madi et al. [4,5,6] have also extensively studied the parasitic infections in migrant workers. They reported the occurrence of parasitic infections in three subsequent years among migrant workers; 33.9% in 2008, 10.2% in 2009 and 21.5% in 2011. In 2008, they recorded intestinal parasitic infections (IPI) amongst food handlers and housemaids from different geographical regions or origin. In 2009, Abu-Madi et al. looked into the trends of IPI among long-term-residents and settled immigrants after introduction of routine albendazole treatment as a condition of entry, residence and issuance of a work permit. Results reported low infection rate (10.2%) with at least one species of intestinal parasite (2.6% with helminthes and 8.0% with protozoan species). While in 2011, Abu-Madi et al. compared IPI between newly arrived and resident workers in Qatar and results showed that 21.5% of the subjects were infected with at least one of the species recorded.

In Malaysia, Suresh et al. [36] conducted a similar study more than a decade ago among migrant workers however, the study only involved clinically ill subjects from University Malaya Medical Centre. The findings of this study provided useful data but the study was not robustly designed to identify priorities for policy recommendations to the health and political authorities. Studies on intestinal parasitic infections have been conducted also among the Malaysian population and infections continue to be a public health problem especially among the poverty- stricken communities. Studies analyzing parasitic infections among various communities in Malaysia include; the Orang Asli (indigenous group) [10,11,37,38,39,40,41], plantation and rural communities [11,42,43,44,45], slum dwellers [46,11], fishing communities [47,48,49,50,51] and flat dwellers [52,53,54,11].

The current study is timely as in the past decade, the number of migrant workers has grown exponentially with a percentage increase of 49% between 2002 (1.06 million) and 2014 (2.07 million) [3]. The global DALY values of intestinal nematode infections particularly due to intestinal obstruction of Ascariasis increase from 0.024 to 0.03 in 2010 (95% uncertain interval: 0.016–0.048) [55]. Despite compulsory medical screening for workers prior to entering the Malaysian workforce, screening for parasitic infections is grossly inadequate or lacking. Therefore, there is an acute need for more accurate and up-to-date information on the parasitic infections in this particular group of workers and an understanding of the factors associated with transmission of these infections, especially as they are likely to impact significantly upon the local community through close contact, lost productivity and the heightened cost of healthcare. The addition of this screening will benefit both the government and employers in particular, due to the improved general health for the worker that further translates to better productivity.

## Materials and Methods

### Subjects

Migrant low skilled and semi-skilled workers can only be employed in Malaysia in five working sectors, namely manufacturing, food services, agriculture and plantation, construction and domestic services. Workers, willing to participate in the current study, were recruited from September 2014 to August 2015 from various agencies and companies around Peninsular Malaysia. A minimum sample size was calculated using a formula by Leedy and Ormrod [56] based on earlier estimates of infection prevalence (36%) values in Malaysia [36]. A total of 388 migrant workers from different categories were recruited.

### Questionnaire

Questionnaires were distributed to participants to gather relevant information related to the study. An individual clinical interview with questionnaire was performed in order to collect individual information including socio-demographic data (nationality, sex, age, religion, marital status, educational level and working sector), migration history (region in country of origin, years of living in Malaysia, mode of entry, working history), environmental health (current residential area, type of accommodation, amenities), life-style habits (smoker, consumer of alcohol and user of illegal drugs), recent episodes of illness (health care utilization, mode of payment, health history) and occupational health and safety (safety hazard, personal protective equipment). In the survey, participants were also questioned on their history of taking anthelminthic drugs. The interview process was performed through an interpreter especially if migrant workers had difficulty in understanding Malay or English. All participants were fully informed of the nature of the study in order to enable maximum co-operation and completion of consent forms.

### Collection and analysis of fecal samples

After consent was obtained and the questionnaire completed, each individual was provided with a plastic container marked with a specific identification number and the name of the participant. The participant was instructed to scoop a thumb size fecal sample into the container, ensuring that the sample was not contaminated with urine. All samples were preserved in 2.5% potassium dichromate solution and brought back to the laboratory at the Institute of Biological Science, Faculty of Science, University of Malaya. For the formalin ether concentration technique, approximately 1 to 2g of sample were mixed with 7 ml of formalin and 3 ml ethyl acetate and centrifuged for 5 minutes at 2500 rpm. After centrifugation, 4 layers were seen, composed of ethyl acetate, debris, formalin and pellets containing parasites. A drop of pellet was taken and stained with Lugol’s iodine on a clean glass slide. The slide was examined under a light microscope at 10x and 40x magnification for helminthes and protozoa, respectively. For *Cryptosporidium* sp., modified Ziehl-Neelsen staining technique was conducted. A smear was made on a glass slide and allowed to dry. Then the smear was fixed with methanol for about 5 minutes and afterwards flooded with cold strong neat carbol fuchsin for 5 to 10 minutes. The slide was washed in tap water and differentiated in 1% acid alcohol until color ceased to leach. The smear was next rinsed under tap water, again followed by counter staining with malachite green for 30 seconds. Slides were blotted dry and examined using 1000x oil immersion objective. Three slides per sample were examined by experienced microscopists and further confirmed by their supervisors in the Department of Parasitology, Faculty of Medicine in the University of Malaya.

### Statistical analysis

Prevalence data (percentage of subjects infected) are shown with 95% confidence limits (CL_95_), as described by Rohlf & Sokal [57] using bespoke software. Prevalences were analyzed using maximum likelihood techniques based on log linear analysis of contingency tables using the software package SPSS (Version 22). Analysis was conducted in two phases. In the first phase, full factorial models were fitted with the intrinsic factors sex (2 levels, males and females), age (5 age classes comprising those <25 years old, 25–34 years old, 35–44 years old, 45–54 years old and those >54 years) and nationality (5 countries, Indonesia, Bangladesh, Myanmar, India and Nepal). Infection was considered as a binary factor (presence/absence of parasites). These explanatory factors were fitted initially to all models that were evaluated. For each level of analysis in turn, beginning with the most complex model, all possible main effects and interactions were investigated and those combinations that did not contribute significantly to explaining variation in the data were eliminated in a stepwise fashion beginning with the highest-level interaction (backward selection procedure). A minimum sufficient model was then obtained, for which the likelihood ratio of χ2 was not significant, indicating that the model was sufficient in explaining the data. The importance of each term (i.e. interactions involving infection) in the final model was assessed by the probability that its exclusion would alter the model significantly and these values relating to interactions including presence/absence of infection are given in the text. The remaining terms in the final model that did not include presence/absence of infections are not given but can be made available by the authors upon request.

In the second phase, models were fitted with four environmental factors (employment sector [Construction, manufacturing, plantation, food services and domestic services], educational level [no formal education, primary education only, education to high school level and to university level], accommodation [hostel/employer provided or own/rented] and years of residency [less than one year or more than 1 year] and presence/absence of infections). The most significant of the intrinsic factors detected in the first phase of the analysis was also included and the model re-run as explained above.

### Ethical considerations

An ethical clearance was obtained from the ethics committee, University Malaya Medical Centre, Malaysia prior to commencement of the study (Reference number: MECID NO: 20143–40). All adults provided written, informed consent to participate in the study and a parent/guardian gave consent on behalf of any child participant. Furthermore, all individual tested positive were notified of their condition through their respected employers.

## Results

### Socio-demographic characteristics

A total of 388 volunteers of migrant workers provided stool specimens. The socio-demographic profile of this subset comprised 304 males (78.4%) and 84 females. Among the males, 37.4% were between 25 to 34 years old (n = 145), 29.4% were younger than 25 (n = 114) and 23.2% older (n = 90 for 35 to 44 years). Most respondents were from Indonesia (n = 167, 43%) followed by Nepal (n = 81, 20.9%), Bangladesh (n = 70, 18%), India (n = 47, 12.1%) and Myanmar (n = 23, 5.9%). The majority were involved in the domestic service sector (n = 105, 27.1%), followed closely by the food service sector (n = 104, 26.8%), while, only a small proportion were from among those working on plantations (n = 71, 18.3%), manufacturing (n = 61, 15.7%) and construction (n = 47, 12.1%) sectors.

### Intrinsic effects on prevalence of intestinal parasitic infections

#### Higher taxa

Stool screening revealed a high proportion of workers positive for intestinal helminthes and protozoan infections (both helminthes and protozoa combined = 62.9% [56.87–68.55]). There was no significant effect of age or sex, but a highly significant effect of nationality was found (*χ*^2^_4_ = 38.1, *P*<0.001). Prevalence was higher among the Nepalese and Indians (Table 1) compared with Indonesians, Bangladeshi and Myanmar. Analyses of combined helminthes infections yielded a similar outcome, with again only nationality showing a significant effect on prevalence (*χ*^2^_4_ = 47.4, *P*<0.001). The highest prevalence was also among the Nepalese and Indians with lower values among the remaining three national groups (Table 1). In contrast to the above, none of the main effects (sex, age or nationality; see Table 1 for nationality) were significant in the case of combined protozoan infections, but there was a weak interaction between sex, age and infection (*χ*^2^_4_ = 10.3, *P* = 0.036). This arose primarily through relatively small differences in prevalence in age class 3 (males = 8.8%, n = 57; females = 24.2%, n = 33), age class 4 (males = 28.6%, n = 21; females = 0%, n = 8), and age class 5 (males = 0.0%, n = 4; females = 33.3%, n = 6), but sample sizes in some subsets were small.

**Table 1:** Prevalence of intestinal parasitic infections amongst migrant workers according to nationality, employment sector, education, accommodation type and years of residence in Malaysia.

			Prevalence (%) ± 95% confidence limits		
Factor	Level	N	All parasites	Combined helminthes	Combined protozoa
Nationality					
	Indonesia	167	52.1 [43.07–61.00]	43.1 [34.48–52.13]	21.6 [15.04–29.78]
	Bangladesh	70	52.9 [41.21–64.15]	45.7 [34.67–57.35]	14.3 [7.85–24.44]
	Myanmar	23	56.5 [36.02–75.34]	43.5 [24.66–63.98]	21.7 [8.99–43.34]
	India	47	83.0 [65.67–92.73]	78.7 [61.18–89.84]	31.9 [17.57–49.59]
	Nepal	81	**84.0 [72.78–91.56]**	**80.2 [68.34–88.64]**	**32.1 [21.46–44.55]**
Employment Sector					
	Construction	47	59.6 [41.80–75.54]	53.2 [35.33–69.61]	12.8 [4.59–28.56]
	Manufacturing	61	**77.0 [66.64–85.19]**	**75.4 [64.99–83.62]**	27.9 [19.08–38.37]
	Plantation workers	71	53.5 [41.80–64.75]	49.3 [37.55–61.04]	11.3 [5.61–20.79]
	Food services	104	74.0 [70.34–82.50]	68.3 [61.30–74.59]	**33.7 [27.28–40.75]**
	Domestic services	105	48.6 [41.41–55.72]	37.1 [30.49–44.28]	24.8 [19.12–31.39]
Educational Level					
	Primary only	166	56.0 [47.04–64.63]	50.0 [41.00–59.00]	19.9 [13.50–27.88]
	High school	160	**69.4 [60.71–76.95]**	61.9 [53.09–69.92]	**30.6 [23.05–39.29]**
	University	8	62.5 [28.93–88.88]	**62.5 [28.93–88.88]**	0 [0–36.46]
	No formal education	54	64.8 [54.51–74.15]	53.7 [43.36–63.57]	18.5 [11.72–27.77]
Accommodation					
	Hostel/ Employer	272	**69.5 [64.69–73.91]**	**63.6 [58.63–68.32]**	**23.9 [19.83–28.46]**
	Own/rented house	116	47.4 [39.97–54.90]	37.1 [30.13–44.54]	23.3 [17.43–30.21]
Years of residence					
	Less than 1 year	134	**79.1 [71.97–85.02]**	**71.6 [63.91–78.30]**	**29.9 [22.97–37.67]**
	More than 1 year	254	54.3 [49.47–59.19]	47.2 [42.37–52.11]	20.5 [16.78–24.69]

#### Individual helminth species

*A*. *lumbricoides*. This was the most common species with an overall prevalence of 43.3% [37.45–49.32]. Prevalence was almost twice as high among males (47.7% [42.39–53.00]) compared with females (27.4% [17.28–39.89]). This was a significant difference when fitted only with infection (*χ*^2^_1_ = 11.5, *P* = 0.001), but when nationality was taken into account (*χ*^2^_4_ = 68.5, *P*<0.001), the effect of host sex disappeared. Prevalence did not differ significantly between different age classes.

Hookworms. The overall prevalence of hookworms was 13.1% [9.56–17.78]. There was no difference between prevalence in male and female subjects (males = 13.2% [9.95–17.19] and females 13.1% [6.51–24.04]), but there was a significant effect of age (*χ*^2^_4_ = 18.8, *P* = 0.001). Prevalence was highest in the youngest age class and none of the ten subjects in the oldest age class was infected (for age classes 1–5, prevalence = 23.7%, 8.3%, 12.2%, 3.4% and 0% respectively). Prevalence did not differ significantly between the 5 nationality classes.

*T*. *trichiura*. This was the rarest of the 3 major intestinal nematode species with a prevalence of 9.5% [6.50–13.72]. There was a marked difference between sexes with prevalence among males (11.5% [8.48–15.35]) being more than 4 times that among females (2.4% [0.33–10.10]), a difference that was highly significant (*χ*^2^_1_ = 11.5, *P* = 0.001). Prevalence also varied significantly between age classes (*χ*^2^_4_ = 13.2, *P* = 0.010), with the highest prevalence among the youngest individuals and no infection recorded among the oldest (for age classes 1–5, prevalence = 17.5%, 8.3%, 3.3%, 6.9% and 0% respectively). With both age and sex taken into account, prevalence also varied significantly between the different nationalities (Table 2, (*χ*^2^_4_ = 13.2, *P* = 0.010)). Prevalence was highest among those from Myanmar and lowest among subjects from India.

**Table 2:** Prevalence of individual helminth species amongst migrant workers according to nationality, employment sector, education, accommodation type and years of residence in Malaysia.

			Prevalence (%) ± 95% confidence limits			
Factor	Level	n	Hookworms	*A*. *lumbricoides*	*T*. *trichiura*	*H*. *nana*
Nationality						
	Indonesia	167	15.0 [9.44–22.47]	26.3 [19.08–34.84]	9.6 [5.39–16.16]	0 [0–3.13]
	Bangladesh	70	4.3 [1.28–11.81]	41.4 [30.42–53.04]	8.6 [3.80–17.41]	2.9 [0.61–9.70]
	Myanmar	23	**17.4 [6.17–38.87]**	17.4 [6.17–38.87]	**26.1 [12.03–47.78]**	0 [0–14.51]
	India	47	12.8 [4.59–28.56]	68.1 [50.41–82.43]	2.1 [0.12–14.67]	2.1 [0.12–14.67]
	Nepal	81	16.0 [8.44–27.22]	**72.8 [60.60–82.71]**	9.9 [4.44–19.94]	**4.9 [1.47–13.63]**
Employment Sector						
	Construction	47	10.6 [3.35–26.30]	36.2 [21.28–53.89]	**21.3 [10.16–38.82]**	0 [0–10.60]
	Manufacturing	61	13.1[7.12–22.05]	**72.1 [61.63–80.92]**	4.9 [1.77–12.06]	**4.9 [1.77–12.06]**
	Plantation workers	71	14.1 [7.72–24.32]	25.4 [16.51–36.67]	15.5 [8.50–25.76]	2.8 [0.58–9.74]
	Food services	104	**14.4 [10.01–20.09]**	58.7 [51.54–65.54]	7.7 [4.65–12.40]	1.9 [0.64–5.11]
	Domestic services	105	12.4 [8.33–17.80]	26.7 [20.76–33.44]	4.8 [2.46–8.75]	0 [0–1.96]
Educational Level						
	Primary only	166	10.8 [6.22–17.74]	36.1 [27.93–45.11]	12.0 [7.30–19.03]	1.2 [0.18–5.12]
	High school	160	13.1 [8.06–20.08]	53.8 [44.94–62.29]	5.6 [2.70–11.08]	**2.5 [0.76–7.00]**
	University	8	**25.0 [4.64–63.53]**	**62.5 [28.93–88.88]**	12.5 [0.64–50.00]	0 [0–36.46]
	No formal education	54	18.5 [11.72–27.77]	31.5 [22.64–41.77]	**13.0 [7.16–21.22]**	1.9 [0.30–7.26]
Accommodation						
	Hostel/ Employer	272	**14.0 [10.79–17.84]**	**49.6 [44.61–54.66]**	**11.4 [8.52–15.01]**	**2.6 [1.37–4.72]**
	Own/rented house	116	11.2 [7.14–16.82]	28.4 [22.16–35.65]	5.2 [2.69–9.53]	0 [0–2.17]
Years of residence						
	Less than 1 year	134	**17.9 [12.49–24.87]**	**58.2 [50.20–65.93]**	**12.7 [8.15–18.91]**	1.5 [0.36–5.03]
	More than 1 year	254	10.6 [7.92–14.03]	35.4 [30.90–40.21]	7.9 [5.60–10.96]	**2.0 [0.98–3.88]**

*H*. *nana*. This species was recorded in just 7 subjects (1.8% [0.72–4.31]) and therefore statistical analysis was not robust. Four subjects were from Nepal, two from Bangladesh, and one from India (Table 2) and no infections were detected among the Indonesians or subjects from Myanmar. All seven infected subjects were male.

*E*. *vermicularis*. Only two cases of *E*. *vermicularis* were detected (0.5% [0.14–2.22], both among male subjects, one from Indonesia and the other from Bangladesh.

#### Individual protozoan species

Three species of intestinal protozoans were recorded.

*E*. *histolytica/dispar*. This was the most common protozoan infecting 45 subjects (11.6% [8.19–16.02]. Prevalence did not vary significantly between age classes or nationalities, but there was a significant difference between the sexes (*χ*^2^_1_ = 5.2, *P* = 0.022). Prevalence was twice as high among female subjects (19.0% [10.83–31.00] compared with males (9.5% [6.81–13.16]).

*Giardia* sp. The overall prevalence of *Giardia* was 10.8% [7.51–15.18]. Prevalence was not affected by host age or nationality, although a marginal significance was found with host age classes (Table 3, (*χ*^2^_4_ = 9.9, *P* = 0.042; for age classes 1–5, prevalence = 14.0%, 13.8%, 5.6%, 3.4% and 0% respectively).

**Table 3:** Prevalence of individual protozoan species amongst migrant workers according to nationality, employment sector, education, accommodation type and years of residence in Malaysia.

			Prevalence (%) ± 95% confidence limits		
Factor	Level	n	*Entamoeba*	*Giardia*	*Cryptosporodium*
Nationality					
	Indonesia	167	**13.8 [8.58–21.12]**	8.4 [4.46–14.88]	1.2 [0.17–5.13]
	Bangladesh	70	2.9 [0.61–9.70]	10.0 [4.82–19.14]	2.9 [0.61–9.70]
	Myanmar	23	13.0 [3.66–32.35]	8.7 [1.57–27.81]	4.3 [0.23–21.25]
	India	47	12.8 [4.59–28.56]	10.6 [3.35–26.30]	**10.6 [3.35–26.30]**
	Nepal	81	13.6 [7.00–24.50]	**17.3 [9.56–28.64]**	2.5 [0.38–10.00]
Employment Sector					
	Construction	47	2.1 [0.12–14.67]	8.5 [2.23–23.41]	2.1 [0.12–14.67]
	Manufacturing	61	11.5 [6.06–20.22]	**14.8 [8.38–24.12]**	3.3 [0.88–9.62]
	Plantation workers	71	1.4 [0.14–7.63]	8.5 [3.69–17.34]	1.4 [0.14–7.63]
	Food services	104	15.4 [10.86–21.19]	13.5 [9.22–19.01]	**7.7 [4.65–12.40]**
	Domestic services	105	**19.0 [13.90–25.38]**	8.6 [5.31–13.52]	0 [0–1.96]
Educational Level					
	Primary only	166	9.0 [4.99–15.57]	9.0 [4.99–15.57]	3.0 [1.00–7.71]
	High school	160	**15.6 [10.15–22.95]**	**13.8 [8.66–20.89]**	**3.1 [1.10–7.76]**
	University	8	0 [0–36.46]	0 [0–36.46]	0 [0–36.46]
	No formal education	54	9.3 [4.69–16.96]	9.3 [4.69–16.96]	3.7 [1.17–9.72]
Accommodation					
	Hostel/ Employer	272	9.6 [6.95–12.97]	**12.1 [9.18–15.81]**	**3.7 [2.16–6.06]**
	Own/rented house	116	**16.4 [11.47–22.62]**	7.8 [4.57–12.79]	1.7 [0.51–5.05]
Years of residence					
	Less than 1 year	134	**13.4 [8.75–19.79]**	**16.4 [11.22–23.18]**	2.2 [0.72–6.15]
	More than 1 year	254	10.6 [7.92–14.03]	7.9 [5.60–10.96]	**3.5 [2.10–5.81]**

*Cryptosporidium* spp. This species was detected in 12 subjects (3.1% [1.55–5.95), and none of the intrinsic factors significantly affected prevalence.

### Extrinsic (environmental) effects on intestinal parasitic infections

#### Higher taxa

With nationality taken into account, the prevalence of all parasitic infections differed between subjects who had resided in Malaysia for less than a year and those who have been there for longer (more than one year; Table 1; *χ*^2^_1_ = 10.7, *P* = 0.001). Prevalence also differed between subjects from different employment sectors (Table 1; *χ*^2^_4_ = 38.1, *P*<0.001), with the highest prevalence among workers in manufacturing and the food service sector and the least in those working in domestic service employment, but there were no significant effects of education or accommodation.

Analysis of combined helminthes infections by 1-way tests fitting only individual factors with infection in turn (see Table 1 for prevalence values for all factors and levels) showed that there were highly significant effects of employment sector (*χ*^2^_4_ = 33.0, *P*<0.001; highest among those in manufacturing sector and least among those employed in the domestic service sector), accommodation type (*χ*^2^_1_ = 23.2, *P*<0.001; higher in those living in hostels or employer provided residences) and years of residence (*χ*^2^_1_ = 21.7, *P*<0.001; higher among those with less than 1 year residence) but not of education level (*χ*^2^_3_ = 4.9, *P* = 0.2). Fitting a full factorial model resulted in a more complex outcome with 4 significant interactions affecting prevalence of combined helminthes infections. The strongest interaction was between education, employment and infection (*χ*^2^_12_ = 25.8, *P* = 0.012). The highest prevalence was among workers in manufacturing and the food service sector and the least in those working in domestic sector (Table 1). However, there were exceptions among the 4 education classes. Thus, for those employed in the food service sector, prevalence was highest if the subjects had no formal education (66.7% [27.14–93.71]), only primary education (82.6% [61.13–93.83]) or university education (80.0% [34.26–98.97]), but among those with high school education, although high for those employed in the food service sector (62.9% [51.26–73.27]), prevalence was higher among those working in manufacturing sector (81.8% [72.5–88.66]). In contrast, there were no cases of helminthes infections among those in manufacturing sector if they had not experienced any formal education, or just primary education, but not surprisingly, sample sizes were very small in these latter categories. The other three interactions were between accommodation type and education (*χ*^2^_3_ = 9.3, *P* = 0.025), nationality and employment sector (*χ*^2^_16_ = 28.6, *P* = 0.026), and years of residence and employment sector (*χ*^2^_4_ = 10.2, *P* = 0.037), but all these were weaker than the former and we did not explore these further.

**Fig 1:**
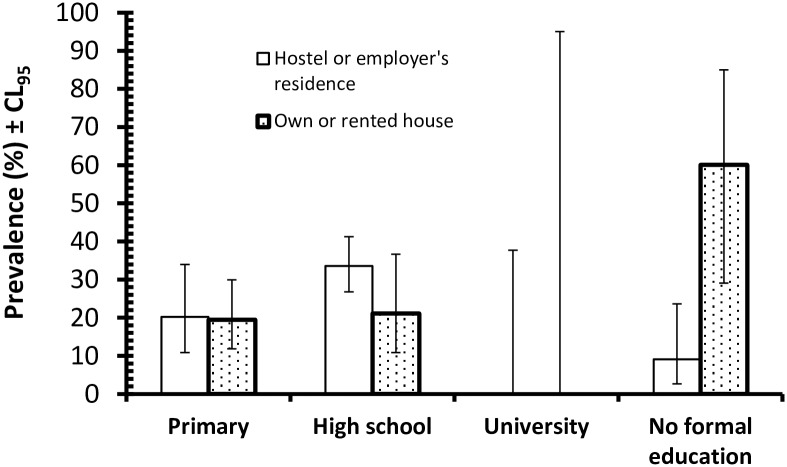
Prevalence of combined protozoan infections in the host population in relation to levels of education and types of residences.

Analysis of combined protozoan infections by tests fitting first just each of the environmental factors with infection in turn as above, (see Table 1 for values for all factors and levels), showed that there were only relatively weak effects, of which the strongest was employment sector (*χ*^2^_4_ = 16.6, *P* = 0.002). Worryingly, prevalence was highest among those in the food service sector and lowest among the plantation workers (Table 1). Prevalence also varied significantly with education (*χ*^2^_3_ = 10.6, *P* = 0.014; highest among those with high school education and lowest among the 8 university graduates) and years of residence in Malaysia (*χ*^2^_1_ = 4.2, *P* = 0.041; higher among those with less than a year of residence in Malaysia). Prevalence did not vary significantly in relation to the accommodation categories. However, when a full factorial model was fitted, thereby controlling for each of the 4 factors above and nationality, these effects were no longer significant and the only term which emerged as significant was an interaction between accommodation, education and infection (*χ*^2^_3_ = 13.8, *P* = 0.003). The principal source of this interaction (Fig 1) was the contrast between subjects whose education ended at either primary or high school levels, among which residence had little effect, and the huge difference in prevalence among those who had no formal education. Among this latter group, those living on their own or rented accommodation (*n* = 10) showed considerably higher prevalence of protozoan infections than those who relied upon their employers to provide accommodation or who lived in hostels (*n* = 44), although the sample size for the former group was low and this needs to be taken into consideration in interpreting the overall significance of this finding.

#### Individual helminth species

*A*. *lumbricoides*. With nationality taken into account, prevalence varied significantly in relation to each of the 4 environmental factors examined (Table 2; years of residence, *χ*^2^_1_ = 18.5, *P*<0.001; Educational level, *χ*^2^_3_ = 14.9, *P* = 0.002; accommodation, *χ*^2^_1_ = 15.3, *P*<0.001; Employment sector, *χ*^2^_4_ = 54.0, *P*<0.001). The highest prevalence was among subjects in manufacturing sector, those living in hostels, residents under one year and perhaps surprisingly among those with a university education, although in the latter case, the sample size was small. There were also several relatively weak more complex interactions with infection (Accommodation x Nationality *χ*^2^_4_ = 9.5, *P* = 0.049, Years resident x Employment sector *χ*^2^_4_ = 10.8, *P* = 0.029, Educational level x Nationality *χ*^2^_12_ = 28.3, *P* = 0.005 and Educational level x Employment sector *χ*^2^_12_ = 24.5, *P* = 0.017) which we did not explore further.

Hookworms. With host age taken into account, none of the environmental factors affected prevalence of hookworms significantly (Table 2).

*T*. *trichiura*. With sex taken into account, the only environmental factors that significantly affected prevalence were employment sector (*χ*^2^_4_ = 19.7, *P* = 0.001) and years of residency (*χ*^2^_1_ = 8.1, *P* = 0.004). Prevalence was highest among those employed in construction and lowest among those in the manufacturing sector (Table 2), and higher among subjects with less than a year’s residency relative to those with more than year’s residency. Accommodation type was marginally significant when fitted on its own with infection (*χ*^2^_1_ = 4.1, *P* = 0.044) but not when other factors were also part of the model.

*H*. *nana*. With just 7 subjects (1.8% [0.72–4.31]) infected by *H*. *nana* statistical analysis was not robust, values for prevalence in each of the different levels of the four environmental factors considered are shown in Table 2.

*E*. *vermicularis*. With only two cases of *E*. *vermicularis* recorded, further analysis was not reliable. Both subjects were registered as not having any formal education, living in hostel accommodation and both with residency exceeding one year.

#### Individual protozoan species

**Fig 2:**
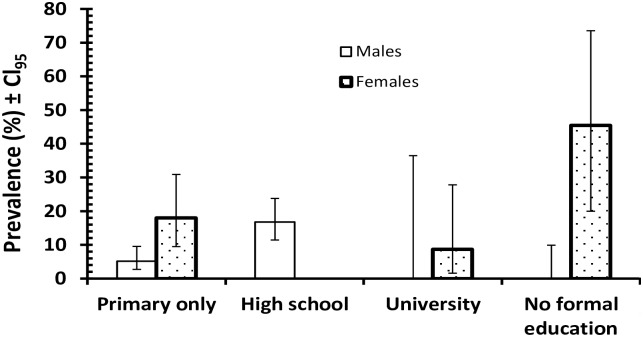
Prevalence of *Entamoeba* in relation to the host-sex and levels of education.

*E*. *histolytica/dispar*. When the four environmental factors were fitted along with host sex and infection, the only significant term was the interaction between sex, education and infection (Fig 2; *χ*^2^_3_ = 18.8, *P*<0.001); education x infection alone was not a significant term in the model. Prevalence also varied with employment sector when employment sector and infection were fitted alone (*χ*^2^_4_ = 23.2, *P*<0.001) but not when the other factors were included in the model.

*Giardia* sp. With host age taken into account, the only environmental factor affecting prevalence was duration of residency (*χ*^2^_1_ = 6.3, *P* = 0.012). As Table 3 shows prevalence of *Giardia* was markedly higher among those with less than one year of residency compared with prevalence among those that have lived locally for more than a year.

*Cryptosporidium* spp. This species was detected in 12 subjects (3.1% [1.55–5.95). We fitted a model with nationality, the 4 environmental factors and infection. The only significant factor to emerge from this analysis was employment sector (Table 3; *χ*^2^_4_ = 12.8, *P* = 0.012). Prevalence was clearly highest among food services sector, and much lower among those in other types of employment, with no infections at all detected among those in the domestic service sector.

## Discussion

The demand for low and semi-skilled workers in several sectors in Malaysia has seen a dramatic rise in the number of workers entering the country from 1.06 million in 2002 to 2.07 million in 2014 [3]. The presence of such a substantial foreign work force originating from countries where parasitic infections are endemic is a major concern especially as this community is highly dynamic, and the emerging and re-emerging infectious diseases that they may carry are a great concern. For the present study we successfully recruited 388 migrant workers from their workplace who provided stool specimens compared to 173 stool specimens of clinically ill subjects from the University Malaya Medical Centre in the previous study [36]. Recruiting workers to participate in the present study was challenging mainly because this screening was not mandatory by FOMEMA (the agency responsible for the implementation, management and supervision for the nationwide mandatory health screening programme for all legal migrant workers), Ministry of Health and Immigration Department of Ministry of Home Affairs upon entry / residing in Malaysia. Other reasons included lack of interest, disgusted with feces and preoccupied with work.

Our study identified a two-fold increase of IPIs (62.9%) among workers compared to a decade ago (36.0%) [36]. Studies reporting analyses of parasitic infections among various communities in Malaysia have been conducted also among the Orang Asli (44.33%-99.2%) [10,11,37,38,39,40,41] plantation and rural communities (32.3%-70.0%) [11,42,43,44,45], slum dwellers (20.6%-90.9%) [46,11], fishing communities (54.2%-98.0%) [47,48,49,50,51] and flat dwellers (5.1%-57.0%) [52,53,54,11]. Our findings based on migrant workers are in agreement with other studies on poverty- stricken communities in Malaysia although some studies have reported fluctuations in prevalence values especially among the slum dwellers (90.9% in 1978 to 20.6% in 2014) [46,11], flat dwellers (57% in 1983 to 5.5% in 2014) [52,11] and rural communities (90.0% in 1970 to 32.3% in 2014) [43,11]. A total of 8 species of parasites were identified (*A*. *lumbricoides*, *T*. *trichiura*, hookworm, *E*. *vermicularis*, *H*. *nana*, *Entamoeba* sp., *Giardia* sp. and *Cryptosporidium* spp.), compared to only 6 species recorded previously (*A*. *lumbricoides*, *T*. *trichiura*, hookworm, *H*. *nana*, *Giardia* sp. and *Blastocystis* sp.) among migrant workers [36]. It is noted that prevalence studies has many limitations and in order to evaluate the impact of IPI on the workforce, further studies are necessary to guide changes in the government policy.

Soil-transmitted helminth (STH) (68.3%) infections were more prevalent compared to protozoan infections (25.5%). Of the three common intestinal nematodes, *A*. *lumbricoides* (43.3%) infections were the most frequently identified, followed by hookworm (13.1%) and *T*. *trichiura* (9.5%). In contrast, a study more than a decade ago highlighted hookworm infections as the most prevalent [36]. However, our result concurs with global findings highlighting *A*. *lumbricoides* infections as the most common helminth among the underprivileged communities [12]. A high presence of *A*. *lumbricoides* eggs contaminating public parks in Peninsular Malaysia has also been reported recently [58].

The demographic profiles of respondents comprised predominantly volunteers from rural areas in their respective countries of origin where IPIs are still very much prevalent and a major concern among the poor and in deprived communities, particularly among workers from India and Nepal where prevalence can exceed 80%. The latest study in the low socio-economic areas of South Chennai, India documented a prevalence of 75.7% with IPI [59], especially in children from rural and urban locations among whom prevalence with *A*. *lumbricoides* ranged between 60 to 91% [60]. This was the most common helminth infection in this community (52.8%). Both studies suggest that inadequate sanitation and poor drainage is likely to have contributed to disease prevalence. Similarly, parasitic infections in Nepal have also been reported as being linked to rapid, unplanned urbanization, open defecation and other unhygienic habits, as well as a lack of health awareness [61, 62, 63]. It is unlikely that workers acquired the infection in Malaysia as all the workers in this study were provided accommodation with adequate facilities such as clean water and flush toilets. It is believed that the infections continue to persist long after entry into the country and during employment and maintained due to bad hygiene practices.

Among the significant explanatory factors associated with the high prevalence of parasitic infections in this country were two main factors i.e, the number of working years in Malaysia and anthelmintic treatment. Workers with an employment history of less than a year or newly arrive workers in Malaysia were those who were most likely to be infected. In addition, they were also most likely to have no history of taking any anthelmintic drugs in the last 12 months. The improvements in health shown by the workers with over a year of residency was possibly due to the impact from better quality of life from the provision of clean water and sanitation. In the event an introduction of anthelminthic treatment is implemented on workers upon entry, this can further reduce infection and improve their overall health. This is not surprising as the mandatory medical screening procedure upon entry to this country excludes examination for IPIs and does not require administration of anthelmintic drugs to newly arrived workers [64]. Therefore our findings call for an improvement in health screening in future to include screening for parasitic infections and compulsory administration of anthelmintic drugs to workers upon entering Malaysia for employment. Such requirement is already implemented in some countries, that depend on an immigrant workforce, as for example in Qatar where currently prospective workers are required to undergo health checks at approved health clinics in their country of origin and if infection with helminths is detected, are routinely given albendazole prior to arrival as a condition for entry, residence and issuance of a work permit [5,6]. Moreover those working in the food service industry have to undergo subsequent annual compulsory examinations by the Medical Commission as a condition of the continuation of their work permits.

Transmission of intestinal nematode infections within the community is predominantly dependent on human behavior, particularly during eating and defecation, personal hygiene, and cleanliness. The high prevalence of parasitic infections among the immigrant community sampled in this study provides an insight into the conditions under which the subjects live, and reflects the availability of environmental sanitation as well as the socioeconomic status of this sector of the population in Malaysia [12]. Despite some of the workers obtaining high education levels, high disease prevalence was still observed amongst the workers possibly acquired due to the lack of sanitation and clean water in their home country compounded with behavioral factor such as bad hygiene practice that continues to persist after entry into the country. Therefore, not only screening is necessary but there is a need for workers to be further educated on good hygiene practices and knowledge of disease transmission.

These findings highlight the urgent need to refine current health polices for Malaysia and especially to include in the future mandatory screening for parasitic infections, as well as STH, of those applying for entry, work permits and residence in Malaysia. Moreover, this should be accompanied by health education campaigns and programs aimed at increasing in the community awareness of the importance of personal hygiene, sanitation, cleanliness and healthy behaviors in controlling parasitic infections [10,11,15].

## Supporting Information

S1 Checklist STROBE Checklist. (DOC)
